# Treatment of juvenile recurrent parotitis with irrigation therapy without anesthesia

**DOI:** 10.1007/s00405-021-06928-w

**Published:** 2021-06-12

**Authors:** Urban W. Geisthoff, Freya Droege, Cathrin Schulze, Richard Birk, Stefan Rudhart, Steffen Maune, Boris A. Stuck, Stephan Hoch

**Affiliations:** 1 Department of Otolaryngology, Head and Neck Surgery, Philipps-University, Marburg, 35043 Baldingerstraße, Germany; 2 Department of Otorhinolaryngology, Essen University Hospital, Essen, Germany; 3 Department of Otorhinolaryngology, Hospitals of the City of Cologne, Cologne, Germany

**Keywords:** Salivary glands, Interventional procedure, Chronic juvenile recurrent parotitis, Minimally invasive therapy

## Abstract

**Purpose:**

No standardized treatment regimen exists for juvenile recurrent parotitis (JRP). The investigators hypothesized that irrigation with saline only without local anesthesia will be an effective and beneficial option.

**Methods:**

Using a retrospective study design, a series of children with typical symptoms of JRP who were treated with at least one irrigation therapy were evaluated. This treatment consisted of irrigation of the affected gland with 3–10 ml saline solution without any type of anesthesia. The outcome variables were patient/parent satisfaction, frequency and duration of acute JRP episodes, and the need for antibiotics before and after irrigation therapy.

**Results:**

The case series was composed of six boys aged 3.3–7.7 years who experienced one to eight sessions of irrigation therapy. The period of follow-up was 9–64 months. We observed a total resolution of symptoms in two children and an improvement in the other four. No relevant side effects were seen.

**Conclusion:**

Our results suggest that irrigation therapy is a reasonable, simple, and minimally invasive treatment alternative for JRP. In contrast to sialendoscopy or sialography, there is no need for general anesthesia or radiation exposure.

## Introduction

Juvenile recurrent parotitis (JRP) is a disorder of unknown origin which affects less than 1% of children [1]. The peak age of symptom onset is 3–6 years and is usually self-limiting by puberty. The disease is more common in boys [2]. Symptoms include predominantly unilateral swelling of the parotid region in combination with redness, pain, difficulties in mastication, and occasional fever (calor, rubor, tumor, and dolor). However, imaging techniques often reveal bilateral involvement [3, 4]. On ultrasonography, multiple hypoechoic inclusions are visible in the swollen parotid gland accompanied by swollen cervical lymph nodes. Secretions of the duct are typically whitish and viscous. Episodes usually last for a few days, but can persist for weeks in rare cases. The duration of asymptomatic intervals between episodes can be several years.

At this time, there is no general consensus about the most suitable treatment regimen for JRP [5]. Antibiotics in combination with analgesics, sialagogues, and gland massage, if tolerated, are often used to cope with episodes. Other methods reported in the literature include irradiation, tympanic neurectomy, ligature of the parotid duct, parotidectomy, and intraductal injection of substances to induce gland atrophy [3]. Interestingly, diagnostic sialography was also found to have a therapeutic effect, which has been attributed to the irrigation effect and potential antibacterial activity of the iodine-based contrast material [6, 7]. This observation led to the therapeutic application of sialendoscopy, thereby avoiding radiation exposure. Similar to sialography, the effect is thought to be due to the irrigation, cleaning and dilatation of the duct system as well as the application of anti-inflammatory solutions, such as cortisone. A growing number of case reports and case series with successful outcomes after sialendoscopy have been reported in the literature. However, the superiority of sialendoscopy compared to other techniques is yet to be statistically proven [8]. In addition, sialendoscopy usually exposes children to general anesthesia and hence, our hesitance in recommending the procedure to affected children. The hypothetical rationale behind both sialography and sialendoscopy as successful treatment methods is the irrigation effect, which appears to break the inflammation cycle by cleaning the salivary duct from mucus plugs and intraductal debris [8]. Therefore, the question arises whether irrigation alone without sialendoscopy might be a suitable treatment option. This less invasive approach avoids both radiation exposure and general anesthesia. However, until now, only few reports have been published about the successful use of irrigation either alone or in combination with topical antibiotics, or systemic steroids as a treatment for various types of chronic sialadenitis, including patients with JRP [3, 9, 10]. In light of the dearth of published studies, the aim of this retrospective study was to analyze the therapeutic effect of irrigation alone on the clinical course of JRP patients. Accordingly, we analyzed outcomes from patients with JRP who were treated by irrigation of the gland with saline solution using a simple flexible intravenous catheter inserted into the papilla of the duct.

## Patients and methods

This retrospective study follows the guidelines of the Helsinki Declaration and the submission of the manuscript has been approved by the institutional review board (“Studienkommission”) of the hospitals of the city of Cologne (28042014). Procedures were performed after obtaining informed consent for the procedures. In this context, all parents of the patients were informed that the offered awake irrigation is an experimental and new therapy and that the level of evidence supporting it is low. As it is no standard but a new therapy, we also had to inform them according to the German juridical situation that "new and until now unknown risks might occur", though this risk is probably low. Parents had to confirm their consent to this with their signature. However, for this type of study, formal consent for study inclusion was not required.

### Technique of the irrigation procedure

**Fig. 1:**
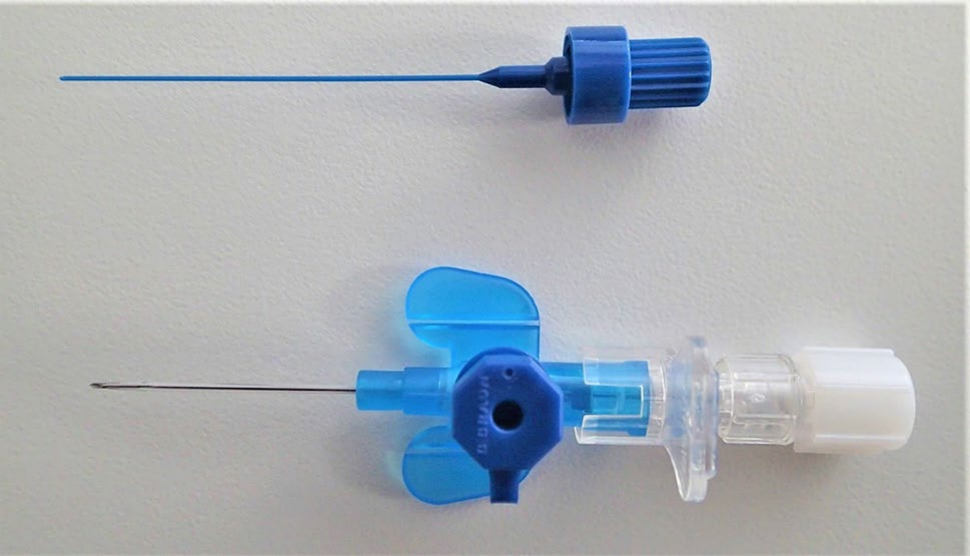
For irrigation an intravenous catheter (22 Gx 1’’ = 0.9 × 25 mm, Vasofix ® Safety, B. Braun Melsungen AG, Melsungen, Germany) was used. The inner steel needle was removed and replaced by a flexible, fitting stylet for closure of the catheter (Mandrin/Stylet, 22G × 25 mm, B. Braun Melsungen AG, Melsungen, Germany)

**Fig. 2:**
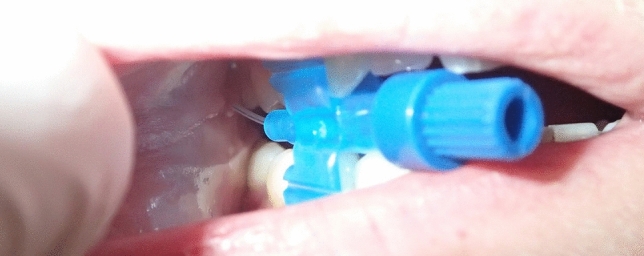
The orifice of Stensen’s duct was identified using a head light and the tip of the catheter was introduced. The patient was asked to bite onto the cap of the Luer lock connector which is situated orthogonally to the main direction of the catheter (photo not from an actual pediatric case but made with an adult volunteer to depict the technique)

All procedures were performed without anesthesia on an outpatient basis. The procedures were performed during asymptomatic periods and never during acute flares of JRP. The steel needle of an intravenous- (IV) catheter (22 Gx 1’’ = 0.9 × 25 mm, Vasofix® Safety, B. Braun Melsungen AG, Melsungen, Germany) was removed and replaced by a flexible, fitting stylet for closure of the catheter (Mandrin/Stylet, 22G × 25 mm, B. Braun Melsungen AG, Melsungen, Germany) (Fig. 1). The stylet stabilized the catheter and made its tip smaller facilitating its introduction into the papilla. Generally, the child sat on the parent’s lap, while the procedure was explained. The child was then asked to open his or her mouth. The orifice of Stensen’s duct was identified using a head light and the tip of the catheter was introduced. The child was asked to bite onto the cap of the Luer lock connector, which is situated orthogonally to the main direction of the catheter (Fig. 2). This held the catheter in place and allowed for easy removal of the stylet. The IV catheter was then connected to a 10 ml syringe filled with saline solution. The solution was then slowly injected into the parotid, initially at a rate of 1–5 ml/10 s. If the child indicated discomfort the injection was paused for 10–20 s and then continued until either all 10 ml were injected or the tolerance of the child was reached. At the end of the injection, an obvious swelling of the gland could be observed. The catheter with the attached syringe was left in place for an additional 5 min. The child was then asked to release the catheter by opening his mouth and the catheter was removed. Immediately following the procedure, the gland was massaged gently, since the region was always sensitive to pain after irrigation. A small amount of whitish secretion could sometimes be observed from the orifice of Stensen’s duct following the procedure. The patient was then requested to maintain a conservative management using sialagogues (e.g. cherry pits or chewing gum) in combination with self-massage of the gland. Patients were recommended to come in for a follow-up evaluation after 3–6 months or earlier if there were any problems. In some instances, the patient was satisfied with the results and requested the irrigation be repeated to further decrease the symptoms.

### Patients

11 boys (age 3.3–11 years; mean age: 6 years) with typical symptoms and findings of JRP were seen at our hospital from September 2007 to February 2014. JRP was diagnosed in the case of at least three acute episodes of parotitis in 1 year and typical ultrasonographic imaging of a parotid gland consisting of multiple hypoechoic areas ("leopard skin" pattern) in combination with cervical lymphadenopathy. We recommended a conservative treatment regime consisting of sialagogues such as cherry pits, chewing gums, olive stones and daily repeated self-massage of the gland for all patients as a basic therapy. Antibiotics (Amoxicillin, Cefaclor) and analgesics (Ibuprofen, Paracetamol) were also recommended for cases of acute exacerbations. In addition to the basic conservative therapy, the following options were discussed: a) sialendoscopy, b) therapeutic sialography, c) irrigation therapy (as described above), or d) no further procedures. After performing the above-mentioned informed consent meetings, the parents of six patients (3.3–7.7 years) decided to proceed with irrigation therapy (c), while the rest continued with basic conservative therapy (d). The ethnic background of those six patients was as follows: four Mediterranean, one Kazakhstan and one German. The right parotid gland was affected clinically in five patients, while one patient suffered from inflammation on the left side. Additionally, two children reported less severe symptoms in the apparently clinically unaffected side. Ultrasonography revealed signs of bilateral chronic inflammation in all six patients.

## Results

**Fig. 3:**
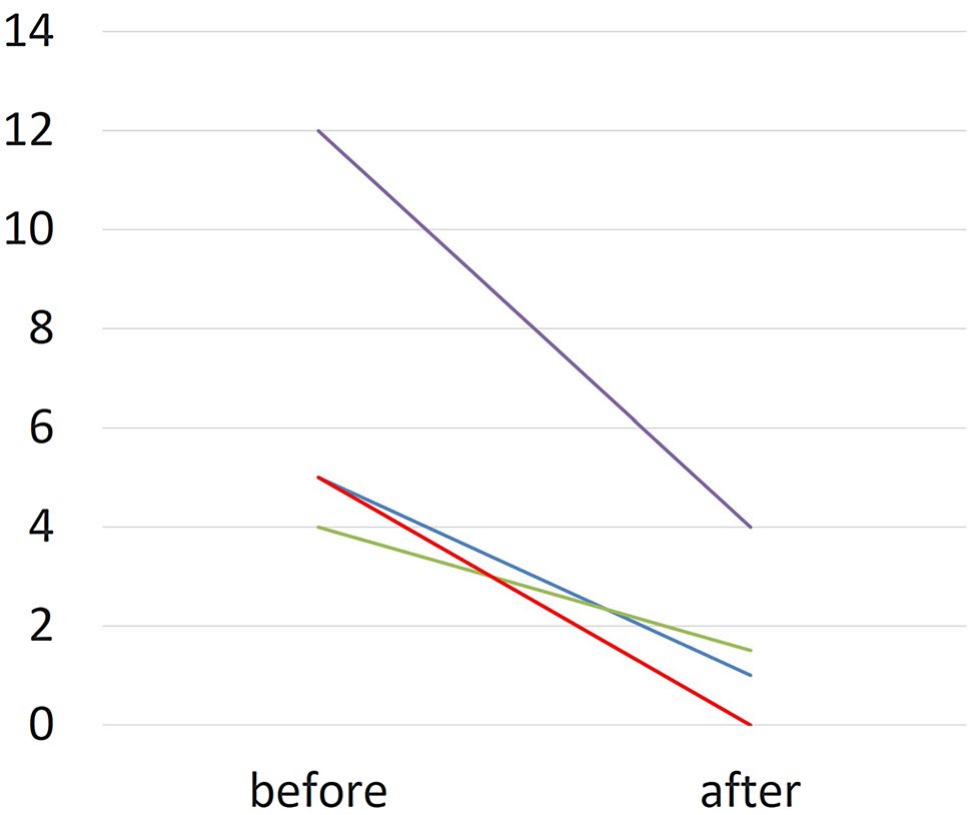
Number of episodes per year before and after the irrigation therapy. The two patients 3 and 6 (3.8 and 7.3 years old) experienced a total cessation after 1 rsp. 2 irrigations (red line). Patient 2 (7.7 years old) suffered from one episode per year for further 3 years after one irrigation procedure (blue line). Patient 4 (4.9 years old) showed between one and two minor swellings per year without the need for antibiotics after two sessions of irrigation. Patient 5 (3 years old) showed a reduction from 12 to 4 episodes per year after one irrigation treatment (violet, line). *The data of patient 1 (5.4 years old), who experienced a total cessation of symptoms after eight treatments are not illustrated here as the parents were not able to remember the exact number of initial episodes

The mean age of the six children receiving irrigation therapy was 5.4 years (Table 1). It should be noted that not all data from patient #1 could be collected. Parents stated that symptoms started at an age range from 1.2 to 6.2 years (mean 3.7 years; *n* = 5). Before therapy, the children suffered from 4 to 12 episodes of acute parotitis per year for which they received antibiotics on most occasions. Irrigation therapy consisted of one irrigation procedure each in three children, two irrigations each in two children and a total of eight irrigations in one child. The volume of saline used per session ranged from 3 to 10 ml. Irrigation caused an immediate swelling of the affected gland which according to the parents returned to normal after 30–90 min. No other side effects were observed. All six children and their parents stated that irrigation therapy led to at least a distinct improvement (*n* = 3) if not total resolution of the symptoms (*n* = 3) (Table 1, Fig. 3). The mean follow-up time after the last irrigation was 27.7 months (range 9–64 months) (Table 1).

**Table 1:** Patients’ results before and after irrigation therapy

Case no	Age at first symptoms (years)	Age at first irrigation (years)	Gland side	Episodes before therapy			Number of irrigation treatments	Volume used for irrigation (ml)	Intervals between irrigations (days)	Length of follow-up after the last irrigation (months)	Episodes after last irrigation			Subjective assessment by the parents
				Frequency (/years)	Duration (days)	Need for antibiotics in case of an episode					Frequency (/years)	Duration (days)	Need for antibiotics in case of an episode	
1	n.k	5,4	R	n.k	n.k	n.k	8	3–9	3–20 *	64	*****	n.k	***	Distinct improvement; free of symptoms during the last year of follow-up
2	5,3	7,7	R	5	7	Nearly always	1	10	–	59	1	14	The first two ones, then none any more	Free of any symptoms for the last 2 years
3	3,1	3,8	R	5	7	Nearly always	2	10	35	12	0	n.a	n.a	Free of any symptoms
4	2,7	4,9	R	4	3	Always	2	5–6	112	10	0/3**	n.a	No	Distinct improvement
5	1,2	3,3	L (B)	12	10	Always	1	5	–	12	4	7	In three of four cases	Distinct improvement
6	6,2	7,3	R (B)	5	5	Always	1	8	–	9	0	n.a	n.a	Free of any symptoms

n.a: not applicable; n.k.: not known; *all eight irrigations took place within 67 days; **mother stated that no major swelling as before occurred, but three minor ones; ***two times each during the first 2 years after treatment, afterwards no need for antibiotics anymore; ****no major swellings after the first 2 years, but seldom minor swellings of parotid and of cervical lymph nodes during colds, no swellings at all during the last year of follow-up; *****at least two major episodes each during the first 2  years, afterwards no further major swelling

## Discussion

To our knowledge this is the first report of successful usage of irrigation therapy with saline alone to specifically treat JRP. Up to now, only a few reports have been published about the successful use of irrigation either alone or in combination with topical antibiotics, or along with systemic steroids as a treatment for chronic sialadenitis in general. In this context, irrigation with tetracycline or erythromycin was suggested by Quinn and Graham in 1972 [9], irrigation in combination with systemic steroids by Baurmash in 2004 [3], and irrigation with saline and with or without penicillin by Antoniades et al. in 2004 [10]. All the authors reported a good response to the irrigation method. However, these case series included a heterogeneous group of patients and thus only a small number of patients with JRP. Hence, Baurmash et al. reported about one 4-year-old boy with JRP, who was treated successfully with irrigation therapy in combination with systemic steroids [3]. The irrigation therapy carried out by the author included sialography, duct dilatation with lacrimal probes, ductal irrigations with sterile saline or neodecadron ophthalmic solution. Neither the specific type of treatment given to the patient nor the usage of anesthesia was specified in the report. Antoniades and co-authors reported a larger case series of 82 patients with various types of chronic sialadenitis treated by intraductal injection of saline with or without penicillin G [10]. Altogether 27 patients suffered from chronic parotitis and ranged from 8 to 65 years of age. At least eight of those patients also had sialolithiasis. Follow-up occurred for 15 of the 27 patients and 14 were proclaimed to be symptom-free between 1 and 14 years after the irrigation therapy. Only three of those 14 patients were treated with saline irrigations without penicillin. Unfortunately, the study by Antoniades et al. does not clearly indicate if the irrigation therapy was successful for patients with JRP, and if penicillin was essential in this group. A further retrospective study by Roby et al. described a reduction in frequency and duration of symptoms with a primary cure rate of 58% in 12 patients with JRP after ductal corticoid irrigation through a catheter [11]. In contrast to the present analysis with a mean follow-up of 27.7 months, the mentioned procedures were always performed under general anesthesia with a comparably short mean follow-up of only 3.8 months. Furthermore, most of the previous studies included sialography in the diagnostic setting before therapy [3, 9, 10], which also proved to have a therapeutic effect. Additionally, the combination of irrigation with an active ingredient such as antibiotics or systemic corticosteroids makes it difficult to determine the efficacy of irrigation treatment alone. However, our observation strongly suggests that irrigation alone, without the use of an active ingredient may be effective, even if this has not been definitively proven.

Sialendoscopy has also been discussed as a treatment for JRP. Some authors reported that even one sialendoscopic session may be sufficient to heal the patient [2, 12–15], while others observed an improvement, and not a cure [16]. The percentage of patients, who are symptom-free after the procedure shows great variety in the literature. Thus, in a retrospective analysis performed by Berlucchi et al., a complete resolution of symptoms could be observed in 35% of the 23 included patients after one session of sialendoscopy with steroid irrigation [13]. Kanerva et al. found 90% of 20 patients to be symptom-free after 1 sialendoscopy and 100% after a second sialendoscopy using isotonic saline solution with 0.1% lidocaine [14]. A meta-analysis performed by Ramakrishna et al., included 7 studies with a total of 120 patients, and revealed an overall primary success rate of 73% [17]. Cleaning of the duct system may be more efficient using sialendoscopy compared to our technique. Using optical control and directly entering parts of the duct system, it is possible to focus the irrigation and the pressure to flush out intraductal debris, or to use other instruments like Dormia baskets for this task. Furthermore, sialendoscopy may be a useful diagnostic tool for the assessment of a specific obstructive etiology for sialadenitis and thus allows for the correct diagnosis of JRP. However, intraoperative findings of a specific obstructive etiology, as stones or ductal stenosis are rare [18]. Furthermore, the effect of the mechanical manipulation by introducing and advancing the endoscope remains unknown. Touching the inflamed duct walls with the relatively sharp tip of the endoscope may have no relevance, but could theoretically lead to scar formation. Canzi et al. summarized ten studies on sialendoscopy and JRP. They stated that potential side effects were possible ductal breech (up to 8%), proximal duct stenosis (up to 66%) and, upper airway obstruction (up to 11%) [19]. In contrast, the intravenous catheter used in the present study was soft and flexible and it was only introduced into the most distal part of the duct. Hence, the risk for an injury of the ductal wall should be even lower than for sialendoscopy. In addition, it must be kept in mind that sialendoscopy in pediatric patients often requires general anesthesia [12, 14, 16, 20–22] depending on the age and compliance of the patient. In this context the results of two studies, performed by Papadopoulou-Alataki et al. [23] and Konstantinidis et al. [20] suggest, that local anesthesia may be useful only for patients older than 8 years. Although the risks of general anesthesia are limited, it is still preferable to avoid them. In particular, the possible neurodevelopmental impact of anesthetic drug exposure in early childhood must be considered [24] Thus, the U.S. Food and Drug Administration pronounced a warning in 2017 for the use of anesthetics in children less than three years of age, especially with regard to repeated applications [25]. Rosbe and co-workers discussed that JRP patients who underwent sialendoscopy had similar outcomes as those with conservative therapy. However, the costs of sialendoscopy were about 45 times higher than conservative management [26].

Even if one assumes that there is enough evidence to perform an irrigation therapy with or without sialendoscopy, a number of questions still remain about the best way to do so. It yet has to be clarified if the irrigation solution should contain corticosteroids, antibiotics, or other additives. Also, different volumes of irrigation fluid, intervals and repetitions have been reported in the literature. Antoniades and co-workers used 1.5–2 ml for the parotid gland and repeated the procedure up to 28 times over the course of 2 years [10]. Quinn and Graham administered 1.5–2.5 ml in children daily for a total of 5 days [9]. Both groups pointed out that the irrigation fluid should be retained within the glands for 5–10 min. Quinn and Graham suggested using local anesthetics to avoid a burning sensation resulting from the irrigation, while Antoniades and colleagues expressed concerns about overfilling of an anesthetized gland [9, 10]. This concern is, however, not justified since the irrigation volume used during sialendoscopy is typically much higher (e.g. as much as 60 ml [2]) and the procedure in children is often performed under general anesthesia without any possibility for feedback. To our knowledge, damages resulting from overfilling have not been reported.

The drawbacks of our study are similar to most other studies in the field such as small study population, retrospective design and lack of a control group. It would have been desirable to use the five cases of non-treated children with JRP as a control group. Unfortunately, a matching was not possible as those five children had a higher age and fewer symptoms. The lack of a control group is especially important as JRP has a tendency of self-healing. Hence, it is difficult to distinguish the therapeutic effect from the natural course of the disease. However, self-healing is usually expected around puberty; the children in this study experienced their improvement well before. Additionally, the parents and researchers in the present study had the impression that the documented clinical improvement always occurred after irrigation therapy. Considering the possible risks of more invasive treatment procedures, which usually have to be performed under general anesthesia, the results indicate that irrigation therapy alone may be an effective and safe alternative that should be considered as part of a treatment plan for JRP.

## Conclusion

Our observations indicate that the use of irrigation therapy with saline only without general or local anesthesia is a viable and less invasive alternative to sialendoscopic or sialographic approaches for the treatment of JRP. Further trials could help determine differences in the efficacy of different methods in comparison to control groups, and to investigate the effect of added substances, such as corticosteroids, antibiotics, and local anesthetics.

## Data Availability

Available upon request.
